# Zinc isotope evidence for sulfate-rich fluid transfer across subduction zones

**DOI:** 10.1038/ncomms13794

**Published:** 2016-12-16

**Authors:** Marie-Laure Pons, Baptiste Debret, Pierre Bouilhol, Adélie Delacour, Helen Williams

**Affiliations:** 1 Department of Earth Sciences, Cambridge University, Downing St, Cambridge CB2 3EQ, UK; 2 Department of Earth Sciences, Durham University, Elvet Hill, Durham DH1 3LE, UK; 3 Université de Lyon, UJM-Saint-Etienne, Laboratoire Magmas et Volcans, UMR 6524, CNRS, UBP, IRD, F-42023 Saint-Etienne, France

## Abstract

Subduction zones modulate the chemical evolution of the Earth's mantle. Water and volatile elements in the slab are released as fluids into the mantle wedge and this process is widely considered to result in the oxidation of the sub-arc mantle. However, the chemical composition and speciation of these fluids, which is critical for the mobility of economically important elements, remain poorly constrained. Sulfur has the potential to act both as oxidizing agent and transport medium. Here we use zinc stable isotopes (δ^66^Zn) in subducted Alpine serpentinites to decipher the chemical properties of slab-derived fluids. We show that the progressive decrease in δ^66^Zn with metamorphic grade is correlated with a decrease in sulfur content. As existing theoretical work predicts that Zn-SO_4_^2−^ complexes preferentially incorporate heavy δ^66^Zn, our results provide strong evidence for the release of oxidized, sulfate-rich, slab serpentinite-derived fluids to the mantle wedge.

## Abstract (web summary)

During subduction, fluids are released into the mantle wedge, but the exact compositions of these fluids are unclear. Pons *et al*. by analysing zinc isotopes from serpentinite provide evidence that oxidized, sulphate rich fluids are released to the mantle wedge during subduction.

Arc lavas erupted at convergent plate boundaries have geochemical signatures that differ from other mantle-derived magmas. They are considered to be more oxidized than mid-ocean-ridge basalts^1^,^2^,^3^,^4^,^5^ and are enriched in fluid-mobile and volatile elements^6^, a geochemical signature that reflects the interaction of mantle wedge peridotites with fluids released by the downgoing slab^3^,^7^. From the ridge to the trench, the oceanic lithosphere experiences hydration and oxidative alteration^8^,^9^ and ultimately delivers volatile element-rich oxidized material to the mantle wedge during subduction^10^,^11^. It has therefore been proposed that slab-derived fluids released during prograde metamorphism can modify the redox state of the mantle wedge^1^,^12^. As water does not readily dissociate under these conditions^2^,^13^, sulfate and carbonate-bearing fluids are considered to be the most likely oxidizing agents^2^,^14^,^15^. Sulfur is considered to be particularly important as one mole of SO_4_^2−^ can oxidize 8 moles of Fe^2+^. However, there is no direct^16^,^17^ evidence of SO_4_^2−^ transfer to the mantle wedge by slab-derived fluids during prograde metamorphism. This is in part because there are few robust geochemical means of tracking sulfate-bearing fluid transfer between the slab and mantle wedge. Recent work on isotope fractionation of Zn complexes^18^,^19^,^20^,^21^ during fluid/rock interaction^22^,^23^ suggests that Zn isotopes have potential as such a tracer, as distinctive Zn isotope effects are observed when Zn is complexed with carbonate and sulfate ligands^18^,^19^,^22^.

In this study, we investigate the nature of slab-derived fluids and trace their transfer to the mantle wedge through a Zn isotope study of Alpine meta-ophiolites and Himalayan ultramafic samples. The studied Alpine ophiolites (Supplementary Fig. 1) sample a paleo-slab and record different stages of prograde, subduction-related metamorphism (from green-schist to eclogite facies)^24^,^25^. They are mostly composed of serpentinites^24^,^25^,^26^ that are formed by the hydration and oxidation of the oceanic lithosphere and can incorporate up to 13% of water^8^, as well as sulfur and chalcophile elements mainly as sulfides^15^,^27^. During subduction, the breakdown of slab serpentine minerals releases fluids and volatiles to the mantle wedge^15^,^26^,^28^. These samples provide a unique natural laboratory to study the chemical impact of slab devolatilization and were used in a recent iron isotope study by Debret *et al*.^29^ to bring constraints on the nature of slab-derived fluids. Debret *et al*.^29^ observed that the Fe isotope compositions of theses serpentinites progressively increased with metamorphic grade as bulk Fe^3+^/∑Fe decreased^29^. These variations were interpreted in terms of the release of isotopically light sulfate rich and/or hypersaline (Cl^−^-rich) fluids to the mantle wedge. These results motivated our study, as Zn isotopes have the potential to distinguish between these two scenarios^18^,^19^ and provide additional mass balance constraints. Zinc isotopes provide subtlety different information to Fe isotopes in that: Zn is not redox-sensitive; Zn is a trace element in the serpentinite sample (∼40 p.p.m.), while Fe is a major element (>8% in the samples^29^); and Fe isotopes are affected by complexation with both sulfate and chlorine ligands while Zn isotopes are not affected by the latter^18^,^19^.

Our results show a striking correlation between Zn isotope compositions and subduction-related metamorphism in the Alpine samples. Serpentinite δ^66^Zn values decrease with increasing metamorphic grade, while fluid-derived materials record heavier Zn isotope compositions. As existing theoretical work predicts the preferential uptake of heavy Zn isotopes when Zn^2+^ forms complexes with sulfates^18^,^19^, this observation, combined with carbon and sulfur content analyses and geochemical modelling, demonstrates the release of oxidized, SO_4_^2−^-rich fluids release during subduction-related slab serpentinite devolatilization that are available to oxidize parts of the mantle wedge and reinforces the findings of Debret *et al*.^29^. We demonstrate that these sulfate-rich fluids constitute efficient vectors for transition metal and chalcophile elements that are concentrated in the sub-arc mantle^30^,^31^. Zinc isotopes are thus a powerful tool that can be used to trace the release of oxidized sulfate-rich slab-derived fluids into to the mantle wedge.

## Results

### Zinc isotope compositions and S contents of Alpine samples

**Figure 1:**
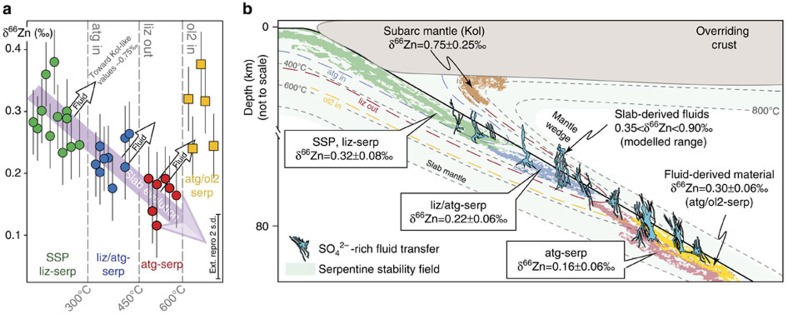
δ^66^Zn evolution of subducted serpentinites and release of a ^66^Zn-enriched slab-derived fluid during subduction. (**a**) Evolution of δ^66^Zn in serpentinites and peridotites from Western Alps ophiolites with respect to mineralogy and prograde metamorphic grade. The purple arrow shows the Zn isotopic evolution of the slab during subduction (serpentinites, circle markers). The white arrows show the inferred release of ^66^Zn-enriched fluids during serpentinite devolatilization associated with the slab subduction and increasing metamorphic grade (atg/ol2-serp, yellow square markers). Sample error bars represent the 2 s.d. reproducibility of replicate analyses and a bold error bar is also shown on all plots indicating the external reproducibility (2 s.d.) of rock standards, from dissolution through to mass spectrometry analysis. Sample abbreviations: SSP, slightly serpentinized peridotites (almost completely unmetamorphosed, green dots), liz (lizardite serpentinites, greenschist facies, green dots), liz/atg (lizardite-antigorite serpentinites, blueschist facies, blue dots), atg (antigorite serpentinites, eclogite facies, red dots); atg/ol2-serp (antigorite and secondary olivine-bearing serpentinites, eclogite facies), Kol: Kohistan Arc gem olivines. (**b**) Schematic diagram illustrating the behaviour of Zn isotopes in serpentinites during subduction and the transfer of a ^66^Zn-enriched sulfate-rich, slab-derived fluid to the mantle wedge. The boxes display the average δ^66^Zn (±2 s.d.) of the slab serpentinites. These means are statistically distinguishable (Student's *t*-test, two-tailed, 95% C.I.). The green, blue and red shaded regions of the subducting slab indicate the hypothetical locations of the studied serpentinite samples in this setting. The yellow shaded regions denote the inferred location of the atg/ol2-serpentinites, and associated veins derived from slab fluids. Light blue fluid patterns: transfer of ^66^Zn-enriched, sulfate-rich fluids across the subduction zone. Grey dashed curves: isotherms. Blue dashed line: appearance of antigorite. Red dashed line: instability limit of the lizardite. Yellow dashed line: appearance of secondary olivine.

To place geochemical constraints on the nature of slab-derived fluids, we analysed fully serpentinized Alpine peridotites^32^ showing the progressive replacement of lizardite (liz), the low temperature form of serpentine, with the high temperature form of serpentine, antigorite (liz/atg and atg-serpentinites, ∼250–400 °C and ∼400–600 °C, respectively, Fig. 1a,b); and the first stages of serpentinite dehydration at eclogite facies where antigorite breaks down to form secondary olivine and chlorite (atg/ol2 ∼650 °C). The atg/ol2 samples consist of serpentinites containing ∼1 mm wide secondary olivine-bearing metamorphic veins and/or shear zones relatively enriched in fluid-mobile elements known to be released during serpentinite devolatilization (for example B, Ba, Li)^26^. Such features have therefore been interpreted as high permeability reaction zones where the fluids released during serpentine phase changes have been localized^26^,^28^. No retrograde phases (for example talc) are observed in any of the studied samples suggesting that they have not been significantly affected by retrograde metamorphism. For reference, we have analysed a series of Alpine slightly serpentinized peridotites (SSP, <20% serpentinization, defined as the replacement of primary minerals by serpentine-group minerals and associated oxides)^32^, and unmetamorphosed liz-serpentinites that are considered to be representative of pre-subduction lithospheric mantle serpentinized on the ocean floor. These samples have high and variable S contents (∼400–1200, p.p.m.; Fig. 3a, Supplementary Table 1), which are likely to reflect heterogeneous S incorporation during initial ocean-floor serpentinization processes^27^,^33^. The Alpine samples show a striking decrease in δ^66^Zn with increasing metamorphic grade (Fig. 1a, Supplementary Fig. 2), with average δ^66^Zn values decreasing from +0.32±0.08‰ (2 s.d.) in oceanic liz-serpentinites to +0.17±0.08‰ in atg-serpentinites with an intermediate mean composition of +0.22±0.06‰ in liz/atg-serpentinites. This slab δ^66^Zn evolution implies a preferential loss of heavy Zn during serpentinite devolatilization and the subsequent release of a heavy-δ^66^Zn fluid. In agreement with this hypothesis, the eclogite facies atg/ol2-serpentinites, which are considered to represent high permeability zones where slab serpentinite-derived fluids are localized^24^,^26^, display heavy δ^66^Zn values with average δ^66^Zn values of +0.30±0.11‰.

### Kohistan Paleo-Island-Arc sample Zn isotope compositions

To extend our study into the general framework of the nature of slab-derived fluids across subduction zones, we have also analysed sub-arc mantle, high temperature (≥450 °C) fluid-derived secondary olivines from the Kohistan Paleo-Island-Arc^30^. These olivines (Kol) are found within veins cross-cutting sub-arc mantle dunites, and are highly enriched in chalcophile elements (for example Zn, Cu)^30^, suggesting the involvement of S-bearing fluids in their formation. While the exact fluid fluxes and starting compositions likely were not identical in the Alpine and Himalayan systems, highly enriched δ^66^Zn values of +0.75±0.25‰ for these sub-arc mantle olivines (Kol; Fig. 1b, Supplementary Fig. 2) broadly mirror the observed Alpine trend.

## Discussion

Although this progressive depletion of serpentinites in ^66^Zn suggests significant Zn isotope fractionation during serpentinite devolatilization, alternative processes must first be considered. These are: Zn isotope fractionation during the initial serpentinization of the oceanic lithosphere; pre-existing protolith Zn isotope heterogeneity; and interaction with sediment-derived fluids^34^. The first scenario can be ruled out as Pons *et al*.^22^ have demonstrated that mid-ocean ridge serpentinization has no substantial effect on the δ^66^Zn values of abyssal serpentinites^22^, which are within error identical to those of oceanic mantle peridotites (0.20–0.35‰). In agreement with this, the Alpine liz-serpentinites and the SSP, which are considered as proxies of pre-subduction oceanic lithosphere^29^,^35^,^36^ display similar δ^66^Zn values ranging from 0.24 to 0.36‰. To address the second issue, we constructed plots of δ^66^Zn and established tracers of serpentinite protolith fertility, such as Mg-number and Al_2_O_3_/SiO_2_ (ref. 22). No correlations were observed between δ^66^Zn and these tracers (Supplementary Table 1 and Supplementary Fig. 3). Interaction with external fluids (third scenario) can also be ruled out as an explanation for the observed variations in δ^66^Zn as there are no correlations between δ^66^Zn and tracers of interaction with sediment-derived components (for example enrichment in fluid-mobile elements such as As, B, Cs, Li, U, Sr, see Supplementary Table 1)^34^,^37^,^38^. Furthermore, crustal melting requires an elevated subduction geotherm^39^, which is inconsistent with the inferred P-T conditions of the Alpine ophiolites studied here^24^,^25^.

The Zn isotope evolution of the Alpine serpentinites with prograde metamorphism must therefore be linked to processes involving the release of ^66^Zn-enriched fluids during subduction-related serpentinite devolatilization. Zinc in serpentinites is present in rock sulfides^22^,^40^ and in the serpentine minerals structure where it replaces magnesium in MgO_6_ octaedredra^41^ (see Methods – geochemical models). During prograde metamorphism, Zn is partially removed from the rock by sulfides leaching and serpentine mineral devolatilization, and the serpentinites lose ∼30% of their initial Zn contents (see Supplementary Table 1 and Methods – geochemical models) into slab-derived fluids. This observed loss of isotopically heavy Zn from the slab is consistent with equilibrium, rather than kinetic stable isotope fractionation as the latter would predict the preferential mobilization of isotopically light Zn, which is not observed. The magnitude and direction of the serpentinite prograde Zn isotope fractionation can be used to place constraints on the speciation and complexation of Zn^2+^ in slab fluids, as Zn stable isotope fractionation at equilibrium is sensitive to the presence of sulfate and carbonate ligands^18^,^19^,^22^. Theoretical work combining *ab initio* structure calculations and statistical mechanics^18^,^19^,^20^,^21^ predicts that both SO_4_^2−^– and CO_3_^2−^–Zn^2+^ complexes will preferentially incorporate isotopically heavy Zn^2+^. Although the precise chemistry of the fluids released during serpentinite dehydration remains uncertain^15^,^42^,^43^, the release of fluids enriched in either SO_4_^2−^ or CO_3_^2–^ can explain the progressive depletion of Alpine serpentinites in heavy Zn.

The release of CO_3_^2−^ with prograde metamorphism has been documented in multiple subduction zones, and is mainly controlled by fluid-mediated carbonate minerals dissolution^14^,^44^,^45^. Interactions between ultramafic mantle rocks and slab-derived CO_3_^2−^-rich fluids have been invoked to explain the negative δ^66^Zn values of the mantle wedge serpentinites from the Mariana forearc mud volcanoes^22^. These processes take place at shallow depth (<15 km^46^) and are associated with widespread carbonate subduction^47^. However, the lizardite to antigorite transition and antigorite breakdown in our Alpine samples occurs at much higher depths, from ∼30 to 45 km and >80 km, respectively^29^ and, as stated earlier, there are no correlations between δ^66^Zn and tracers of interactions with sediment-derived components such as carbon-bearing fluids^34^,^37^,^38^. In addition, our liz-serpentinite samples, which are representative of pre-subduction oceanic lithosphere, have low total carbon contents (see Supplementary Table 1, Fig. 3c, ∼100–1000, p.p.m.) as do the higher metamorphic grade samples and correlations between serpentinite δ^66^Zn and carbon content are absent (Fig. 3c). These observations suggest that carbon is not the dominant metasomatic agent in these specific Alpine serpentinites and that the carbonate contents of the fluid(s) released during serpentinite devolatization are too low for significant complexation of slab Zn with CO_3_^2−^ ligands. As neither Cl^−^ nor HS^−^ species have been demonstrated to cause significant Zn isotope fractionation^18^,^19^, the most likely candidate is therefore SO_4_^2−^.

**Figure 2:**
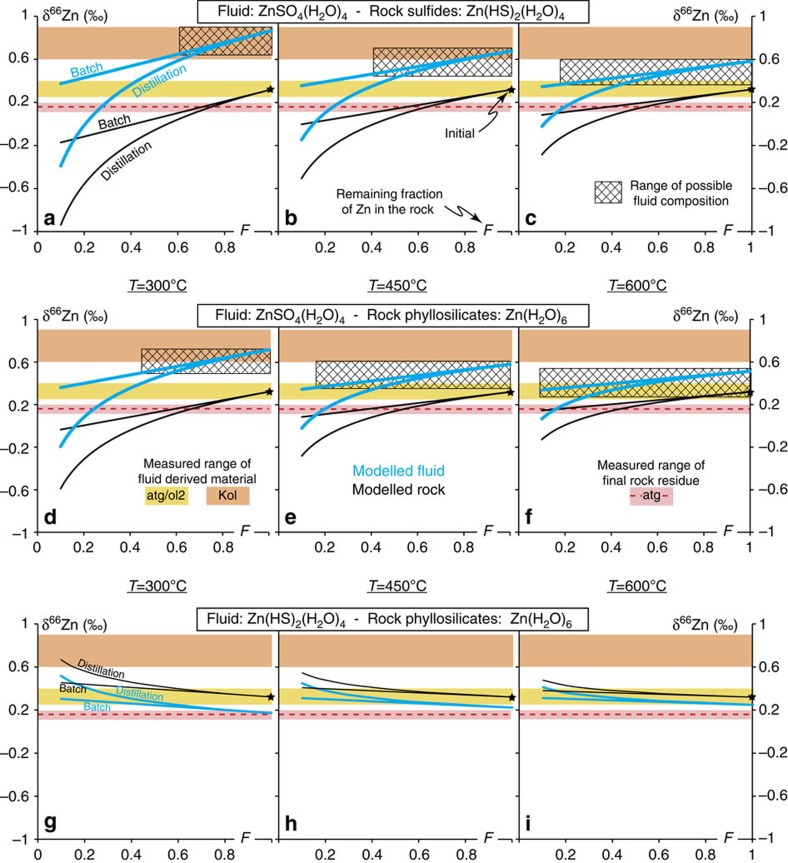
Modelling of δ^66^Zn evolution in slab fluids and residual serpentinites for the sulfate- and sulfide-bearing fluid scenarios. Evolution of residual serpentinite δ^66^Zn (in black) and that of associated fluids (in light blue) with the remaining fraction of Zn in the rock (F) using batch and Rayleigh distillation models. The models were performed at 300 °C (**a**,**d**,**g**), 450 °C (**b**,**e**,**h**) and 600 °C (**c**,**f**,**i**). The models were performed using fractionation at equilibrium fractionation factors, **a**,**b**,**c**: between Zn contained in the serpentinite sulfides (ZnHS_2_(H_2_O)_4_) and a sulfate-rich fluid (ZnSO_4_(H_2_O)_4_). **d**,**e**,**f**: between Zn contained in the serpentinite phyllosilicates (Zn(H_2_O)_6_) and a sulfate-rich fluid (ZnSO_4_(H_2_O)_4_) and **g**,**h**,**i**: between Zn contained in the serpentinite phyllosilicates (Zn(H_2_O)_6_) and a sulfide-rich fluid (ZnHS_2_(H_2_O)_4_). The fate of Zn in the rock sulfides is not reported as there is no fractionation at equilibrium with only one species in solution (ZnHS_2_(H_2_O)_4_). The solid coloured areas represent the range of δ^66^Zn in measured samples for antigorite Alpine serpentinites (in red), and fluid-derived material (in yellow: atg/ol2-serpentinites and in orange: Kohistan olivines Kol). The grid represents the range of possible fluid compositions from the initial starting composition (black star) to the end of the antigorite field, defined by the observed range of δ^66^Zn in the samples (in red). Fractionation factors from Black *et al*. and Fujii *et al*.^2,14^. The model is fully described in the method.

**Figure 3:**
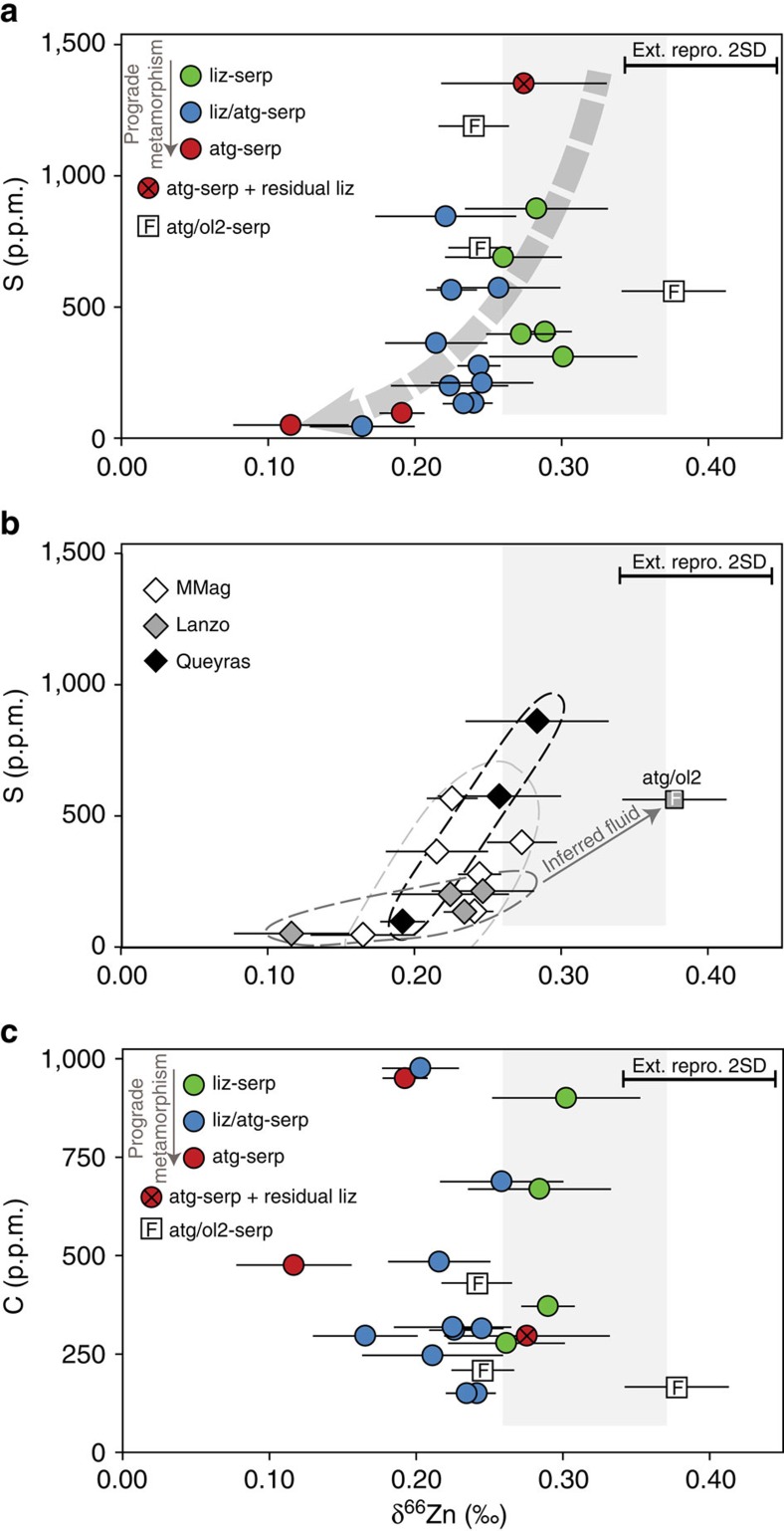
Zinc isotope compositions versus sulfur and carbon contents in serpentinite samples from various Western Alps ophiolites. (**a**) Evolution of bulk δ^66^Zn and S content in Western Alps serpentinites samples with regard to their main mineralogy and metamorphic grade. (**b**) Evolution of bulk δ^66^Zn and S content in Western Alps serpentinites samples with regard to their geographical location. The depletion in heavy Zn isotopes is associated with the loss of S from the slab during prograde metamorphism and serpentinite devolatilization (schematic grey dotted arrow; a). Secondary olivine-bearing serpentinites (white squares) display both heavy Zn values and high S concentrations. This evolution reflects the progressive release of S with metamorphism and the concomitant preferential leaching of heavy Zn isotopes and provides strong evidence in favour of the release of SO_4_^2−^-rich, high-δ^66^Zn oxidizing fluids during serpentinite devolatilization in subduction. At the outcrop scale, this trend is even stronger (b, grey to black dashed envelopes). (**c**) Evolution of δ^66^Zn and C content in Western Alps serpentinites samples with regard to their main mineralogy. No correlation between δ^66^Zn and C content is observed. MMag: Monte Maggiore. liz, atg: lizardite, antigorite-serpentinites. atg/ol2: secondary olivine-bearing serpentinites. Grey fields: expected range of possible S (C) contents and δ^66^Zn compositions of greenschist facies serpentinites, according to the literature^27^,^33^. Sample error bars represent the 2 s.d. reproducibility of replicate analyses and a bold error bar is also shown on all plots indicating the external reproducibility (2 s.d.) of rock standards, from dissolution through to mass spectrometry analysis.

To quantify the heavy Zn depletion of serpentinites with prograde metamorphism and test models of SO_4_^2−^ bearing fluid release, we used *ab initio* calculated fractionation factors^18^,^19^ to model the δ^66^Zn evolution of both residual serpentinites and the released fluids, as sampled by the atg/ol2-serpentinites and Kohistan olivines (Fig. 2, see methods). We considered both batch and distillation models involving chlorine-, sulfide- and sulfate-rich fluids. The release of sulfide (HS^−^) (Fig. 2g–i) and chlorine-rich (Supplementary Fig. 4) fluids cannot explain our data, as the former would drive the residual serpentinites towards high δ^66^Zn values (Fig. 2a–f), which are not observed, whereas in a chlorine-rich environment a loss of >75% of the original serpentinite Zn content at T<300 °C is required to match the observed atg-serpentinite δ^66^Zn values. The latter scenario is unrealistic as Zn concentrations indicate a maximum loss of ∼30% Zn between liz- and atg-serpentinites through lizardite breakdown at T>400 °C. However, models involving sulfate-rich fluids can reproduce the measured range in serpentinite ^66^Zn-depletion with prograde metamorphism (Fig. 2a–f). Critically, the predicted δ^66^Zn values for the fluid released during the serpentinite devolatilization also overlap with the δ^66^Zn values of Kohistan olivines (for T≤500 °C) and of Alpine ol2/atg-serpentinites (for T°≥450 °C) while the loss of Zn from the initial serpentinite varies from ∼20 to ∼50% (at 300 and 600 °C, respectively), matching the observed decrease in Zn concentration. This striking correspondence between the model predictions and our Zn isotope and concentration measurements provides strong evidence that sulfates are the main Zn ligands in slab-derived fluids in the Alpine subduction system and that secondary olivines in both the Alpine slab and the Kohistan sub-arc mantle record these fluids. This is consistent with the observation that the S contents of these samples decrease during prograde metamorphism from greenschist to eclogite facies by a factor of 10–100 (ref. 15). Sample δ^66^Zn values also decrease with this fall in S content with increasing metamorphic grade (Fig. 3a; Supplementary Table 1), and this is also consistent with the release of S during prograde metamorphism and serpentinite devolatilization. This coupled decrease in serpentinite δ^66^Zn and S content is also present at the outcrop scale (Fig. 3b), where both δ^66^Zn and S decrease with increasing degree of prograde metamorphism (for example δ^66^Zn=+0.25‰, [S]=225 p.p.m., liz/atg-serpentinite and δ^66^Zn=+0.13‰, [S]=50 p.p.m., atg-serpentinite; Lanzo ophiolite). Significantly, secondary olivine-bearing serpentinites display both heavy Zn values and high S concentrations (for example δ^66^Zn=+0.37‰, [S]=560 p.p.m., Lanzo atg/ol2-serpentinite, Fig. 3a,b), providing strong evidence in favour of the release of SO_4_^2−^-rich, high-δ^66^Zn oxidizing fluids during serpentinite devolatilization in subduction^18^,^19^,^22^.

Zinc stable isotopes are therefore a powerful tool to investigate the recycling of Zn into the Earth's mantle, the release and fate of slab-derived fluids in subduction zones and compelling evidence for sulfate transfer from the slab to the mantle wedge (Fig. 1b). The Zn isotope fractionation models presented above (Fig. 2) and the Zn concentration data (Supplementary Table 1) obtained for serpentinite samples suggests that between 50 and 80% of serpentinite Zn is recycled into the mantle. Furthermore, our results allow us to quantify the amount of sulfur needed to form Zn sulfate complexes (see methods) and hence the minimum quantity of S released from the slab. A loss of ∼10 p.p.m. of S from the subducted serpentinites (<2% of its original S content) is enough to account for the Zn transfer from the slab to the fluid. This value is small compared to the measured loss of ∼85–97% of original serpentinite S content during prograde metamorphism, which suggests that considerable excess sulfate is released during serpentinite dehydration. This sulfate will be available to oxidize regions of the supra-subduction zone mantle and will complex with other ions, especially divalent metal cations (for example Cu)^48^, facilitating the transfer of these economically important elements from the subducting slab to the mantle wedge.

## Methods

### Alpine samples description

The selected samples correspond to well-characterized peridotites and serpentinites^24^,^25^,^26^,^49^ from various Western Alps ophiolites recording different metamorphic conditions during subduction (Supplementary Fig. 1). Alpine meta-ophiolites belong to the Ligurian-Piedmont Ocean that was closed by subduction during late Cretaceous and early Tertiary. These remnants of the Ligurian-Piedmont oceanic lithosphere have been formed and hydrated in magma poor settings^50^ and then metamorphosed at various P-T conditions during subduction. They are mostly composed of serpentinites intruded by gabbroic pods and topped by basalts and/or sediments and preserve different steps of serpentinization and deserpentinization^32^. The initial ocean-floor serpentinization is extensively preserved in greenschist alpine meta-ophiolites where serpentinites display lizardite, the low-temperature form of serpentine (liz-serpentinites). During subduction, the prograde destabilization of oceanic lizardite into antigorite occurs from greenschist to blueschist facies^25^, at T∼250–400 °C (liz/atg-serpentinites). At eclogite facies, the serpentinites do not display the textures typical of ocean-floor serpentinization and are fully recrystallized into antigorite (atg-serpentinites, T>400 °C). In these massifs, the incipient deserpentinization of antigorite is marked by the crystallization of olivine (^+^/_−_ chlorite) in metamorphic veins and shear zones (atg/ol2-serpentinites). These correspond to high permeability reaction zones where the fluids released during subduction have been localized^49^.

### Zinc chemical extraction and purification

The Zn extraction and purification chemical procedure is adapted from Moynier *et al*.^51^. Sample digestion: after crushing in an agate mortar, 50–100 mg of powdered samples were digested for 72 h in a 7:3 ml concentrated HF:HNO_3_ mixture at about 130 °C and then evaporated to dryness. Concentrated HCl (5 ml) was added to the dried sample to get rid of fluorides. Once evaporated again, samples were dissolved in 1.5 N HBr and evaporated to dryness. Zinc chemical separation: Teflon columns were filled with 500 μl AG1-X4 100–200 mesh anionic resin stored in 0.5 N HNO_3_. Resin was washed three times with Milli-Q water (18 MΩ-grade) and 0.5 N HNO_3_ and then conditioned with 1.5 N HBr. Sample is loaded onto the column in a 1.5 N HBr medium (Moynier *et al*.)^51^. Zn^2+^ is strongly adsorbed on the resin whereas the matrix elements are eluted. Zinc is then recovered in 0.5 N HNO_3_. The protocol is then repeated to purify the zinc fraction. Because ion-exchange resins fractionate Zn isotopes, full yields are required. By analysing all the recovered fractions, we were able to demonstrate that the yield was better than 99%. The total procedural blank including sample dissolution, chemical purification steps and mass spectrometry measurements was <10 ng of Zn, which represents less than 0.3% of the total sample signal.

### Zinc isotope composition analysis

Zinc isotope ratios have been measured using the Thermo Scientific Neptune Plus multicollector inductively coupled plasma mass spectrometer (MC-ICPMS) at Durham University following the procedure described in Maréchal *et al*.^52^. Isotope ratios are expressed in δ units, where δ is the deviation relative to the standard JMC 3-0749 L (JMC_Lyon_) in permil:


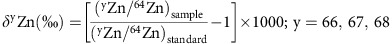


The samples were run in wet plasma mode and introduced by free aspiration in 0.05 N distilled HNO_3_ using a PFA microflow nebulizer (uptake rate: 50 μl min^−1^) and a glass cyclonic spray chamber. Samples were run at 750 ppb Zn, for an average sensitivity of ∼15 V per p.p.m. of Zn. Zinc isotopes (M=64, 66, 67 and 68) were measured together with copper (Cu: M=63, 65). Nickel (Ni) was also monitored at mass 62 to correct the Ni contribution on mass 64. The instrumental mass fractionation was corrected using Cu-doping and sample-standard bracketing^8^. We used a pure Alpha Aesar Cu solution for doping and an exponential law to correct the instrumental mass bias. Samples were bracketed with standards, randomized, and the measurements replicated. Two rock standards (basalts BHVO-2 and BCR-2) were also digested, chemically processed and analysed to compare with literature data, and two pure Zn solution standards were also measured (‘London Zn' and ‘Romil Zn'). Our data (see Supplementary Table 2) are in perfect agreement with previously published values^53^ (Moeller *et al*. and references therein). The long-term reproducibility on δ^66^Zn based on repeated measurements of an in-house Zn standard (Alpha Aesar pure Zn solution, *n*=220) is 0.035‰ (2 s.d.). The total external reproducibility of the chemical and analytical procedure on δ^66^Zn based on repeated analysis of an international rock standard (BCR-2, *n*=9) is 0.06‰ (2 s.d.).

### Sulfur and carbon content analyses

Nancy measurements: whole rock sulfur contents were determined on a Carbon-Sulfur (CS) analyser Leco SC144 DRPC by the Service d'Analyse des Roches et des Minéraux (SARM), at the Centre de Recherches Pétrographiques et Géochimiques (CRPG) in Nancy. This involved introducing 250 mg of powdered whole rock sample into the CS analyser via a ceramic crucible followed by high temperature combustion and infrared detection. The detection limit of the analyses was 0.01%.

Saint-Étienne measurements: sulfur and carbon contents were determined on bulk rock powders using an Elementar Vario Micro Cube coupled, in a continuous flow mode, with an Isoprime Micromass mass spectrometer, at the Laboratoire Magmas et Volcans, Saint-Etienne (France). About 4–40 mg of bulk rock powder were weighed in tin capsules. Tungsten trioxide catalyst was added to each sample powder in the tin capsule to enhance combustion. Calibration of the carbon and sulfur contents was provided using various amounts of sulfanilic acid standard. Detection limit for reproducible carbon and sulfur contents are, respectively, of 20 and 50 p.p.m., and relative precision of carbon and sulfur contents are within 10%.

### Geochemical models

Our measurements show the progressive depletion of ^66^Zn in the bulk rock serpentinites with prograde metamorphism, and the inferred release of a ^66^Zn-enriched fluid during serpentinite devolatilization. Here we considered two types of models to estimate the amount and the Zn isotope compositions in the released fluids. We first considered a batch model where equilibrium between the fluid and the rock is maintained until the reaction is complete, at which time the fluid escapes the serpentinite. We then performed a Rayleigh distillation model in which the fluid leaves the serpentinites as it is formed. In both models we considered that the reaction is finished when δ^66^Zn_rock_=0.17‰, which is the mean value of atg-serpentinites (red dotted lines in Fig. 2, main text, and Supplementary Fig. 4) and we used the mean values for liz-serpentinites for the initial rock value: δ^66^Zn_initial rock_=0.32‰.

In the batch model, the rock δ^66^Zn evolution is calculated following equation (1):


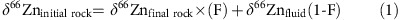


where F is the fraction of Zn that remains in the rock and α_r-f_ is the fractionation factor between the Zn dominant species in the rock phyllosilicates or sulfides and the dominant species in the fluid.

The isotopic composition of the final rock is related to that of the fluid by the equation (2):


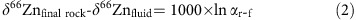


The Rayleigh distillation model can be calculated following equation (3):


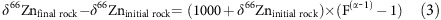


where F is the fraction of Zn that remains in the rock and α=α_f-r_ is the fractionation factor between the dominant species in the fluid and the Zn dominant species in the rock phyllosilicates or sulphides.

Both models were run using fractionation factors calculated for a broad range of temperatures representative of the Ligurian oceanic lithosphere subduction P-T path (see Fig. 1b). The results were plotted for the following temperatures: 300, 450 and 600 °C (see Fig. 2, main text, and Supplementary Fig. 4), which correspond roughly to the end of the liz-serpentinite, liz/atg-serpentinite and atg-serpentinite stability fields, respectively (Fig. 1b). To our knowledge, there are no solid to aqueous zinc isotope fractionation factors determined for serpentinites during subduction. We therefore made the assumption that Zn in phyllosilicate structures is best represented by Zn(H_2_O)_6_, for which theoretical data are available. Zinc in phyllosilicate structures replaces magnesium in MgO_6_ octahedra, where it is bound by six oxygens (ZnO_6_)^41^. In Zn(H_2_O)_6_, Zn is also in an octahedral site^54^ and primarily interacts with the oxygen atoms of the water molecules, which makes it similar to (ZnO_6_). We used Zn(HS)_2_(H_2_O)_4_, the main Zn complex in oceanic serpentinization fluids^19^ to characterize Zn in rock sulfides. Both models were then tested for chlorine-, sulfide- and sulfate-rich fluids, using fractionation factors calculated for ZnCl(H_2_O)_5_, Zn(HS)_2_(H_2_O)_4_ and ZnSO_4_(H_2_O)_4_ ligands, respectively. Results for sulfate-rich and sulfide-rich fluids are presented in Fig. 2 (main text) and results for chlorine-rich fluids are plotted in the Supplementary Fig. 2. All the fractionation factors were calculated using Black *et al*. and Fujii *et al*. data^18^,^19^.

### Serpentinite Zn contents and limit on F values

The average Zn content in liz-serpentinites and SSP (Supplementary Table 1) is ∼43 p.p.m. The average Zn content in atg-serpentinites is ∼35 p.p.m. These two values cannot be directly compared to one another as the initial liz-serpentinites experience a loss of ∼13% water and compaction during prograde metamorphism. The Zn initial (final) content of 1g of liz-(atg-) serpentinites expressed in moles is: n_ini_ (n_final_).


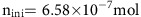



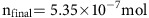


Considering the initial serpentinite loss of water, 1 g of liz-serpentinite corresponds to ∼0.85 g of atg-serpentinite. The corrected value for n_final_ is thus:


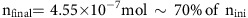


During prograde metamorphism, the serpentinites lose ∼30% of their initial Zn contents. Taking the local Zn content variability into account, we considered values of F comprised between 0.2 and 0.5 as reasonable.

### Sulfates needed to form Zn complex in slab-derived fluids

For 1 g of subducted liz-serpentinite, ∼2 × 10^−7^ mol of Zn is released in the fluid. To form 1 mol of ZnSO_4_(H_2_O)_4_ complex, 1 mol of S is required. The subduction of 1 g of liz-serpentinite consummates ∼6.5 μg of sulfur to form Zn complexes.

### Data availability

The authors declare that the data supporting the findings of this study are available within the article and its Supplementary Information files.

## Additional information

**How to cite this article**: Pons, M.-L. *et al*. Zinc isotope evidence for sulfate-rich fluid transfer across subduction zones. *Nat. Commun.* **7**, 13794 doi: 10.1038/ncomms13794 (2016).

**Publisher's note:** Springer Nature remains neutral with regard to jurisdictional claims in published maps and institutional affiliations.

## Supplementary Material

Supplementary Information Supplementary Figures 1-4, Supplementary Tables 1-2 and Supplementary References.
