# Supplementary material for: Zinc isotope evidence for sulfate-rich fluid transfer across subduction zones
Source: Nat Commun. 2016 Dec 16;7:13794. doi: 10.1038/ncomms13794 (PMC5171646; doi:10.1038/ncomms13794)
Supplement: Supplementary Information — Supplementary Figures 1-4, Supplementary Tables 1-2 and Supplementary References. [file ncomms13794-s1.pdf]

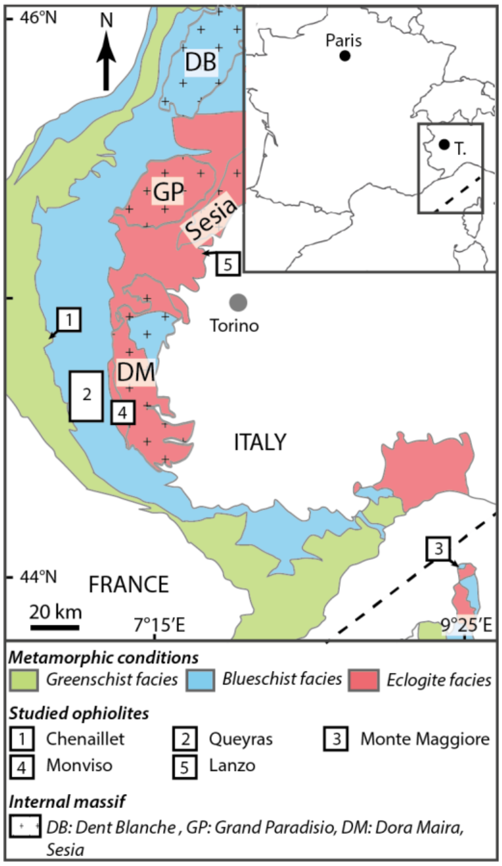

Supplementary Figure 1 | Alpine samples geographic origin. Simplified metamorphic map of the Western Alps showing the spatial distribution of the studied ultramafic ophiolites (numbered white squares).

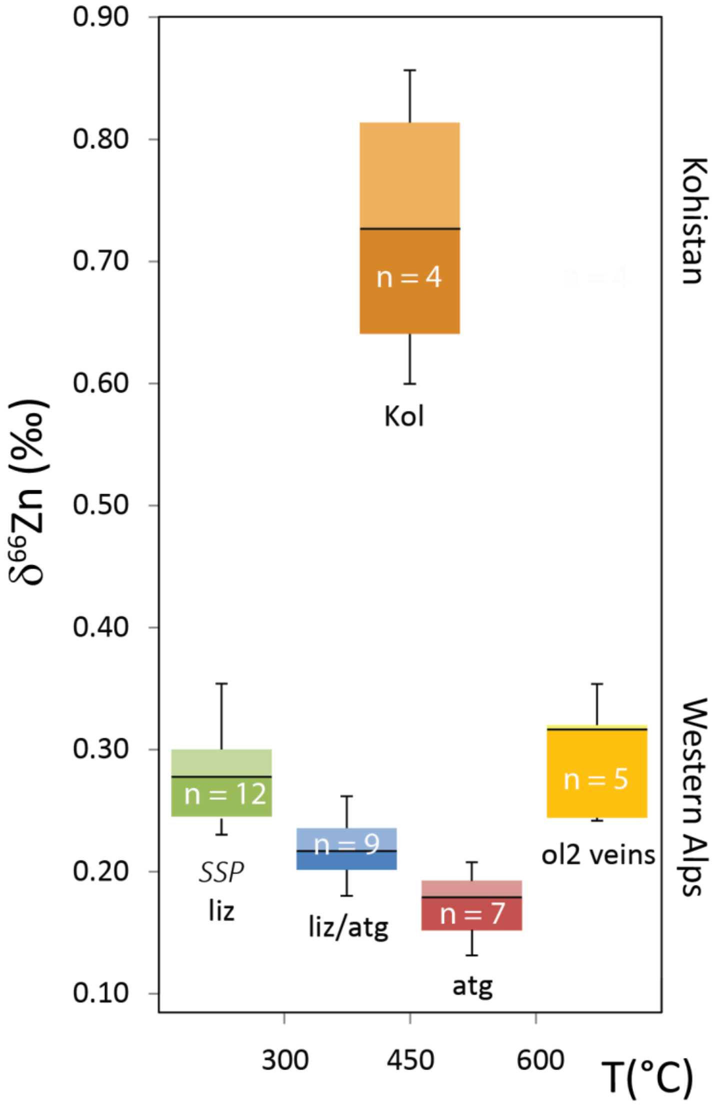

Supplementary Figure 2 | Evolution of $\delta^{66}Zn$ in various serpentinites and peridotites from Western Alps ophiolites samples and Kohistan arc olivines with prograde metamorphism (as temperature). The box represents the 25th–75th percentiles (with the median as a bold horizontal line) and the error bars show the 10th–90th percentiles. SSP: slightly serpentinized peridotites. liz, atg: lizardite, antigorite-serpentinites. atg/ol2: secondary olivine-bearing serpentinites. Kol: Kohistan arc olivines.

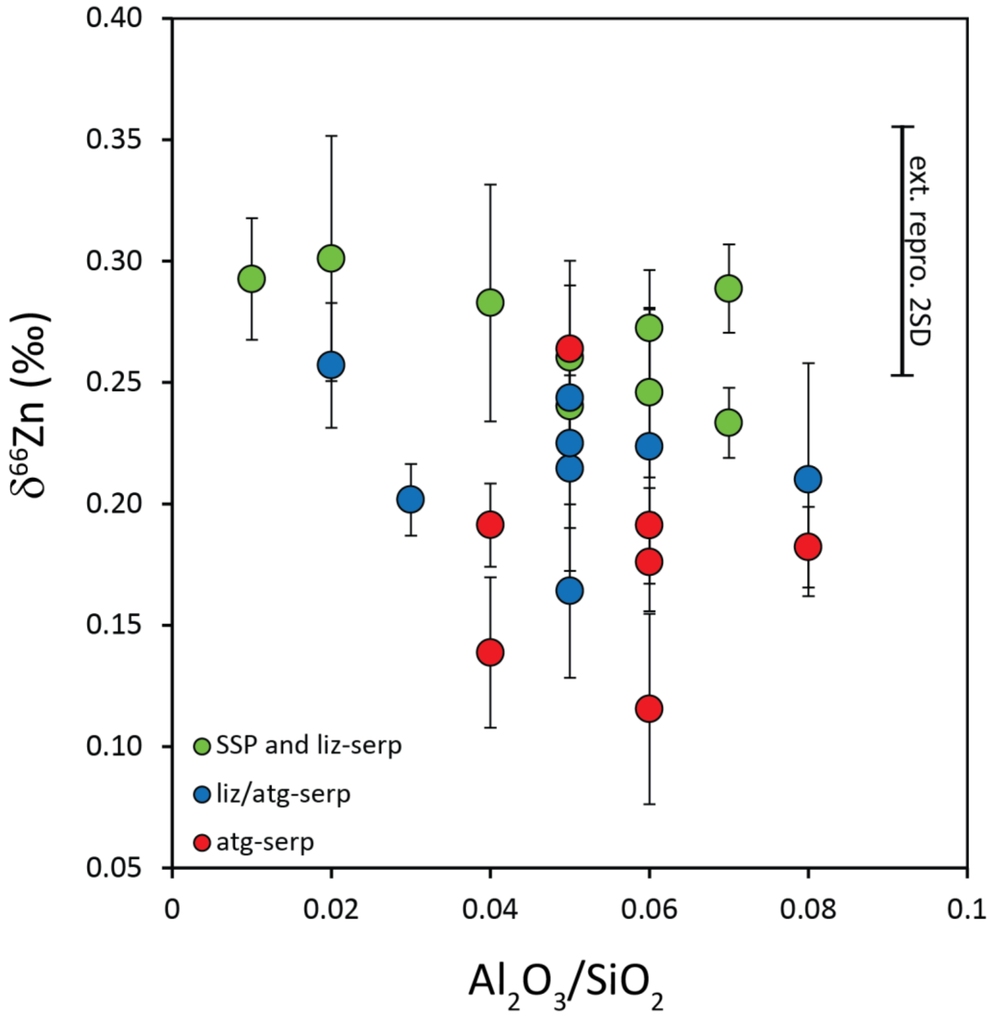

Supplementary Figure 3 | Evolution of δ^{66}Zn in Alpine samples as a function of peridotite fertility. Zinc isotope compositions of Alpine SSP and liz-serpentinites (green circles) and subducted liz/atg- (blue circles) and atg- (red circles) serpentinites plotted against Al_2O_3/SiO_2. There is no correlation between the δ^{66}Zn of a serpentinite and its protolith fertility. liz: lizardite; atg: antigorite. SSP: Slightly serpentinized peridotite. Sample error bars represent the 2 S.D. reproducibility of replicate analyses and a bold error bar is also shown on all plots indicating the external reproducibility (2 S.D.) of rock standards, from dissolution through to mass spectrometry analysis.

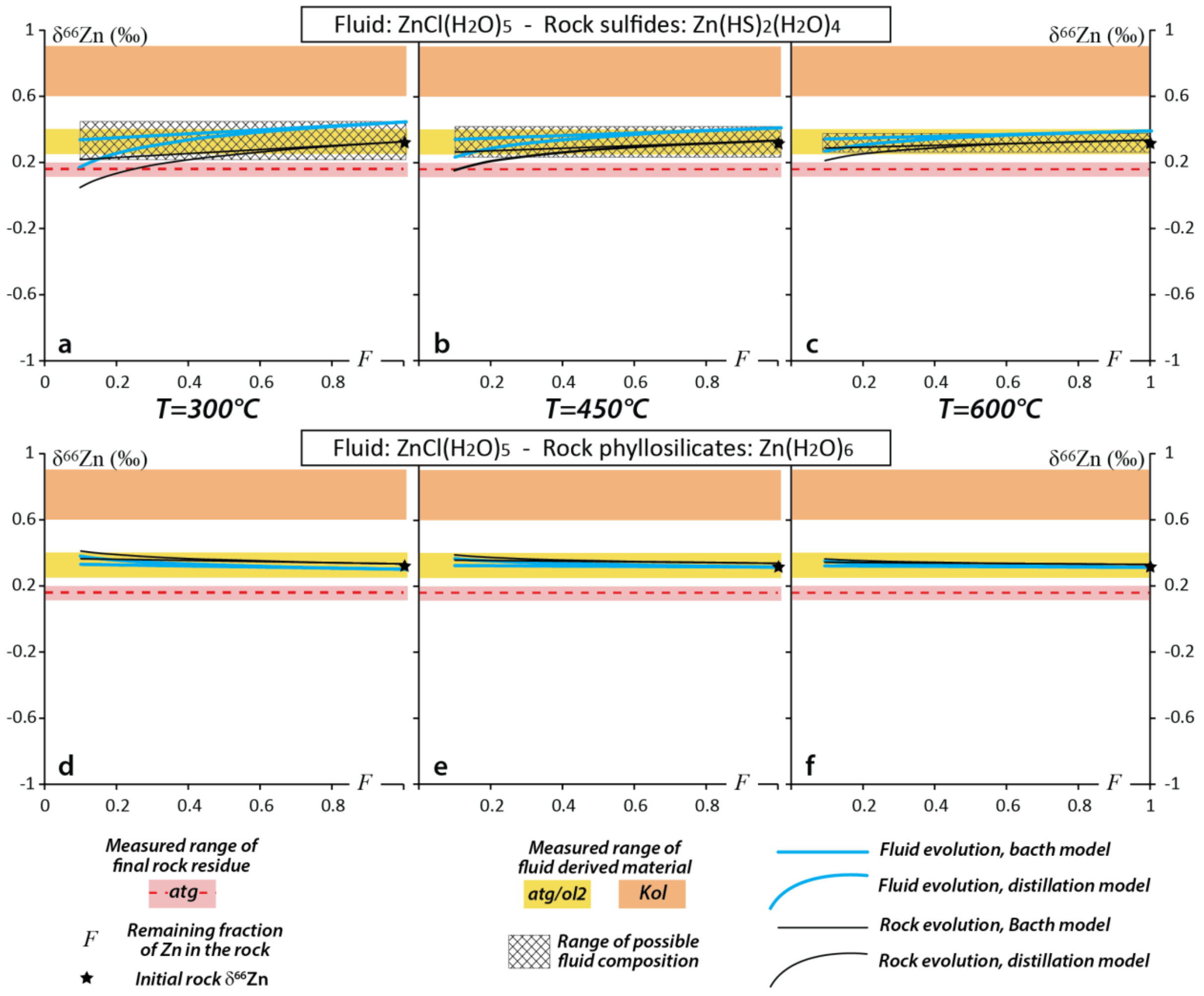

Supplementary Figure 4 | Modelling of the δ^{66}Zn evolution in serpentinites and Zn isotope composition of the fluid released during subduction in a chlorine-rich scenario. Serpentinite δ^{66}Zn evolution (in black) and δ^{66}Zn of the associated released fluid (in light blue) with the remaining fraction of Zn in the rock (F) using a batch model (straight lines) and a Rayleigh distillation model (curves). The models were performed at 300°C (**a** and **d**), 450°C (**b** and **e**), and 600°C (**c** and **f**), using fractionation at equilibrium fractionation factors, **a, b, c**: between Zn contained in the serpentinite sulfides ($ZnHS_2(H_2O)_4$) and a chlorine-rich fluid ($ZnCl(H_2O)_5$). **d, e, f**: between Zn contained in the serpentinite phyllosilicates ($Zn(H_2O)_6$) and a chlorine-rich fluid ($ZnCl(H_2O)_5$). The solid coloured areas represent the range of δ^{66}Zn in measured samples for antigorite Alpine serpentinites (in red), and fluid-derived material (in yellow: atg/ol2-serpentinites and in orange: Kohistan olivines Kol). The grid represents the range of possible fluid composition, released during the rock dehydration, from its initial composition (black star) to the end of the antigorite field (in red). The model is fully described in the method section of the main text.

Sample	Serp. Mineral	Mg#	Al_2O_3/SiO_2	**Zn (ppm)**	Li (ppm)	B (ppm)	As (ppm)	Sr (ppm)	Cs (ppm)	U (ppb)	**δ^{66}Zn (‰)**	2sd	N	S (ppm) St-Étienne	2sd	N	S (ppm) Nancy	**S (ppm) average**	**C (ppm)**	2sd	N
Alpine samples																					
Sligthly Serpentinized peridotite																					
Mag26	-	0.84	0.05	**46**	1.62	-	0.60	0.69	5.38	1.86	**0.24**	0.01	6	169	50	5	100	**135**	**264**	22	2
Mag33	-	0.84	0.06	**41**	2.05	-	0.57	8.09	5.71	17.72	**0.27**	0.02	6	396	-	1	400	**398**	**96**	33	2
LZ19	-	0.83	0.07	**48**	0.80	-	0.05	5.44	1.86	0.38	**0.23**	0.01	4	166	10	3	100	**133**	**226**	132	2
LZ7	-	0.88	0.01	**39**	0.61	-	0.04	1.00	13.37	0.75	**0.29**	0.03	4	-	-	-	-	-	-	-	-
LZ17a	-	0.83	0.06	**43**	2.83	-	0.05	1.28	12.75	0.43	**0.25**	0.03	4	224	5	2	200	**212**	-	-	-
Liz-Serpentinite																					
ch A1*	Liz	-	-	**47**	-	-	-	-	-	-	**0.38**	0.05	2	-	-	-	-	-	-	-	-
ch A2*	Liz	-	-	**45**	-	-	-	-	-	-	**0.36**	0.05	2	-	-	-	-	-	-	-	-
ch A3*	Liz	-	-	**49**	-	-	-	-	-	-	**0.30**	0.05	2	-	-	-	-	-	-	-	-
BCh10	Liz	0.84	0.05	**36**	0.51	13	0.46	1.20	8.44	0.41	**0.26**	0.04	4	680	-	1	700	**690**	**277**	46	2
ICh02	Liz	0.84	0.02	**41**	0.27	-	0.22	1.34	6.34	8.78	**0.30**	0.05	4	322	-	-	300	**311**	**900**	243	2
RQ30	Liz±Atg	0.82	0.04	**37**	2.11	-	0.14	4.61	246.28	-	**0.28**	0.05	4	819	44	2	900	**860**	**669**	231	2
BCh6	Liz±Atg	0.84	0.07	**35**	1.50	14	0.46	5.63	26.15	0.89	**0.29**	0.02	4	412	-	1	400	**406**	**371**	0	2
Atg/Liz-serpentinite																					
BCH9	Liz/Atg	0.83	0.08	**34**	0.81	27	0.53	3.55	5.00	2.12	**0.21**	0.05	4	869	-	1	900	**884**	**246**	37	2
ch ML 4	Liz/Atg	-	-	**31**		-	-	-	-	-	**0.18**	0.03	2	-	-	-	-	-	-	-	-
RQ23	Liz/Atg	0.81	0.02	**56**	0.48	-	0.90	2.04	647.04	3.56	**0.26**	0.04	4	547	-	1	600	**574**	**688**	271	2
MM8	Liz/Atg	0.81	0.05	**32**	0.55	24	0.50	0.94	27.52	0.54	**0.21**	0.03	4	325	-	1	400	**363**	**484**	62	2
MM19	Atg±Liz	0.83	0.05	**31**	0.86	2	0.53	8.57	38.42	40.57	**0.22**	0.02	4	530	-	1	600	**565**	**310**	24	2
MM15	Liz/Atg	0.84	0.05	**51**	0.59	15	0.53	1.38	4.04	3.17	**0.24**	0.01	6	253	19	5	300	**276**	**315**	36	2
RQ01	Atg±Liz	0.85	0.03	**44**	0.05	10	3.97	0.30	8.43	2.95	**0.20**	0.03	4	114	-	1	80	**97**	**976**	214	2
LZ26c	Atg±Liz	0.88	0.06	**38**	0.08	-	0.03	0.23	0.29	0.30	**0.22**	0.04	4	-	-	-	200	**200**	**318**	27	2
MM2	Atg	0.84	0.05	**37**	2.89	-	0.87	1.31	43.10	1.98	**0.16**	0.04	4	40	9	2	50	**45**	**296**	1	2
Atg-serpentinite																					
Vis1	Atg±Liz	0.83	0.05	**29**	0.14	-	0.06	0.53	0.36	0.76	**0.26**	0.06	4	1404	54	4	1300	**1352**	**296**	43	2
LZ14b	Atg	0.83	0.06	**28**	0.00	-	0.09	0.29	6.09	6.00	**0.12**	0.04	4	50	-	1	50	**50**	**475**	85	2
LZ30	Atg	0.88	0.06	**43**	0.01	-	1.23	0.06	0.65	0.43	**0.19**	0.02	6	-	-	-		**96**	**950**	27	2
LZ34d	Atg	0.85	0.08	**36**	0.45	-	0.05	9.99	23.78	0.99	**0.18**	0.02	6	-	-	-	-	-	-	-	-
LZ8b	Atg	0.81	0.06	**46**	0.05	-	0.04	0.54	8.69	0.74	**0.18**	0.02	6	-	-	-	-	-	-	-	-
Vis9	Atg	0.82	0.04	**24**	0.07	-	0.05	0.17	4.77	0.95	**0.14**	0.03	6	-	-	-	-	-	-	-	-
Vis1F	Atg	0.88	0.04	**37**	0.01	-	0.06	0.15	0.38	0.61	**0.19**	0.02	6	-	-	-	-	-	-	-	-

Atg/Ol2-serpentinites																					
Vis5b	Atg+Ol2	0.83	0.03	**23**	0.02	-	0.03	0.22	0.36	0.32	**0.24**	0.02	4	1180	-	1	1200	**1190**	**430**	54	2
RO1	Atg+Ol2	0.83	0.07	**50**	0.16	-	0.13	1.82	62.43	1.10	**0.24**	0.02	4	652	-	1	800	**726**	**208**	22	2
Vis12	Atg+Ol2	0.85	0.03	**39**	0.19	-	0.07	2.52	1.00	1.21	**0.32**	0.03	6	-	-	-	-	-	-	-	-
LZ5	Atg+Ol2	0.83	0.05	**39**	2.00	-	0.06	6.08	18.45	1.41	**0.32**	0.03	6	-	-	-	-	-	-	-	-
LZ27a	Ol2/Atg	0.84	0.06	**35**	1.14	-	0.04	7.41	9.49	0.93	**0.38**	0.04	4	560	-	1	-	**560**	**166**	1	2
Kohistan gem olivines																					
OG1		-	-	**152**	-	-	-	-	-	-	**0.66**	0.06	4	-	-	-	-	-	-	-	-
OG		-	-	**16**	-	-	-	-	-	-	**0.79**	0.04	4	-	-	-	-	-	-	-	-
OGM		-	-	**17**	-	-	-	-	-	-	**0.89**	0.05	4	-	-	-	-	-	-	-	-
Ogi		-	-	**139**	-	-	-	-	-	-	**0.57**	0.05	4	-	-	-	-	-	-	-	-

Supplementary Table 1 | Zinc isotope compositions and S and C concentrations of Alpine serpentines and Kohistan gem olivines

Zinc isotope compositions measured on a ThermoScientific Neptune Plus MC-ICPMS at Durham University. *Data from Pons et al. (2011)[1]. S Nancy: Sulfur contents measured on a Carbon-Sulfur analyser Leco SC144 DRPC at SARM, CRPG in Nancy. S and C St-Étienne: Sulfur and Carbon contents measured an Elementar Vario Micro Cube coupled, in a continuous flow mode, with an Isoprime Micromass mass spectrometer, at the Laboratoire Magmas et Volcans, St-Étienne. Major and trace elements data are from Debret et al. (2013)[2].

Supplementary Table 2 | Zinc isotope standards measurements

Standards		$\delta^{66}Zn_{JMC\ Lyon}$	2sd	n
**Rock**				
**BCR-2**	**Our study**	**0.27**	**0.06**	**9**
	Moeller et al. (2012)	0.33	0.13	3
	Archer and Vance (2004)	0.20	0.09	12
	Cloquet et al. (2006)	0.32	0.13	8
	Chapman et al. (2006)	0.29	0.12	2
	Toutain et al. (2008)	0.26	0.04	5
	Sonke et al. (2008)	0.25	0.04	4
	Hoerzog et al. (2009)	0.33	0.09	
**BHVO-2**	**Our study**	**0.32**	**0.05**	**5**
	Moeller et al. (2012)	0.48	0.13	3
	Hoerzog et al. (2009)	0.29	0.09	
**Pure Zn solution**				
**'London Zn'**	**Our study**	**0.16**	**0.05**	**4**
	Moeller et al. (2012)	0.14	0.06	10
	Arnold et al. (2010)	0.10	0.06	9
**'Romil Zn'**	**Our study**	**-9.06**	**0.04**	**4**
	Moeller et al. (2012)	-9.10	0.07	10
	Chapman et al. (2006)	-9.14	0.08	21

Modified after Moeller et al., 2012[3].

Supplementary references

1 Pons, M.-L. *et al.* Early Archean serpentine mud volcanoes at Isua, Greenland, as a niche for early life. *Proceedings of the National Academy of Sciences* **108**, 17639-17643, doi:10.1073/pnas.1108061108 (2011).

2 Debret, B. *et al.* Trace element behavior during serpentinization/de-serpentinization of an eclogitized oceanic lithosphere: A LA-ICPMS study of the Lanzo ultramafic massif (Western Alps). *Chemical Geology* **357**, 117-133, doi:http://dx.doi.org/10.1016/j.chemgeo.2013.08.025 (2013).

3 Moeller, K., Schoenberg, R., Pedersen, R.-B., Weiss, D. & Dong, S. Calibration of the New Certified Reference Materials ERM-AE633 and ERM-AE647 for Copper and IRMM-3702 for Zinc Isotope Amount Ratio Determinations. *Geostandards and Geoanalytical Research* **36**, 177-199, doi:10.1111/j.1751-908X.2011.00153.x (2012).
